# Physical rehabilitation for older patients with acute HFpEF (REHAB-HFpEF) trial: Design and rationale

**DOI:** 10.1016/j.ahj.2026.107420

**Published:** 2026-03-10

**Authors:** Amy M. Pastva, Gordon R. Reeves, David J. Whellan, Robert J. Mentz, Haiying Chen, Alain G. Bertoni, Pamela W. Duncan, Mark A. Espeland, Shelby D. Reed, M. Benjamin Nelson, Christopher M. O’Connor, Dalane W. Kitzman

**Affiliations:** a Department of Orthopedic Surgery (Physical Therapy Division), Medicine, and Population Health Sciences, Duke University School of Medicine, Durham, NC,; b Claude D. Pepper Older Americans Independence Center, Duke University School of Medicine, Durham, NC,; c Department of Medicine, University of North Carolina School of Medicine, Chapel Hill, NC,; d Department of Medicine, Thomas Jefferson University, Philadelphia, PA,; e Department of Medicine, Cardiology Division, Duke University School of Medicine, Durham, NC,; f Department of Biostatistics and Data Science, Wake Forest University School of Medicine, Winston-Salem, NC,; g Department of Medicine, Section of Internal Medicine, Wake Forest School of Medicine, Winston-Salem, NC,; h Department of Neurology, Wake Forest University School of Medicine, Winston-Salem, NC,; i Department of Population Health Sciences, Duke University School of Medicine, Durham, NC,; j Department of Cardiovascular Medicine, Wake Forest University School of Medicine, Winston-Salem, NC,; k Inova Heart and Vascular Institute, Fairfax, VA

## Abstract

**Rationale:**

Older patients hospitalized for acute decompensated heart failure with preserved ejection fraction (HFpEF) experience persistently poor outcomes, including physical disability, cognitive impairment, depression, impaired health-related quality of life (HRQOL), rehospitalizations, loss of independence, and mortality. In our previous phase 2 REHAB-HF trial, an innovative physical rehabilitation intervention, delivered across three phases–inpatient, 12-week outpatient, and 3-month home-based maintenance–produced large improvements in physical function and HRQOL, with signals of reduced clinical events among patients with acute HFpEF. These findings provide a compelling rationale for a definitive, event-powered trial to evaluate clinical outcomes.

**Hypothesis:**

Targeting physical frailty and multisystem functional impairments using the REHAB-HF intervention–a transitional, tailored, structured, and progressive multidomain rehabilitation program focused on balance, mobility, strength, and endurance–will reduce clinical events in older, predominantly frail patients hospitalized for acute HFpEF.

**Design:**

REHAB-HFpEF is a multicenter, randomized, single-blind, attention-controlled phase 3 trial across 22 U.S. health system centers, enrolling 880 patients aged ≥60 years hospitalized for acute HFpEF. The primary endpoint is combined all-cause rehospitalizations and mortality at 6 months. Key secondary endpoints include major mobility disability (defined as inability to walk ≥160 meters on the 6-minute walk test) and HRQOL measured by the Kansas City Cardiomyopathy Questionnaire. Healthcare costs will also be assessed.

**Conclusions:**

REHAB-HFpEF is designed to address care gaps for frail older adults with acute HFpEF, who currently lack an evidence-based rehabilitation pathway. If successful in meeting endpoints, this trial could establish a scalable rehabilitation intervention that improves recovery, reduces adverse events, and lowers healthcare costs. Findings may shift HF management paradigms, inform guidelines, and influence national coverage policy for this growing, high-risk population.

**Current Status:**

Enrollment ongoing, 478 of 880 (66%) as of 24Feb2027.

**Trial registration:**

Clinicaltrials.gov ID NCT05525663, https://clinicaltrials.gov/study/NCT05525663?term=NCT05525663&rank=1

Acute decompensated heart failure (ADHF) is the most frequent Medicare discharge diagnosis, accounting for over 1.2 million hospitalizations annually and more than $39 billion in healthcare expenditures.^1^ Older adults (≥60 years) hospitalized with ADHF exhibit severe functional impairments, frailty, depression, poor health-related quality of life (HRQOL), and markedly elevated risks of rehospitalizations, loss of independence, and mortality.^1,2^ Despite substantial efforts, most pharmacologic therapies and care coordination models have failed to improve outcomes in this high-risk population.^3–7^ Hospitalization itself, and the accompanying immobility, further exacerbate physical and cognitive vulnerabilities, leaving older adults especially susceptible to disability and rehospitalization.^8^

Our research program was among the first to demonstrate that impairments in physical function are a central, modifiable, and often overlooked contributor to persistent adverse outcomes in older adults with ADHF.^9–14^ In the phase 2 *Rehabilitation Therapy in Older Acute Heart Failure Patients (REHAB-HF)* (*N* = 349), we tested an innovative, structured, progressive, multidomain physical rehabilitation tailored to the severe functional deficits observed in this population. The intervention—initiated during hospitalization and transitioned across outpatient and home-based settings—used an individualized sequencing strategy that prioritized strength, balance, and mobility before endurance to optimize safety and effectiveness in frail older adults.^4,15,16^ The REHAB-HF intervention produced large, clinically meaningful improvements in physical function, frailty, depressive symptoms, and HRQOL, with the greatest benefits observed among the most impaired participants. Although the study was not powered for clinical event differences, numerically lower rates of both cardiovascular and noncardiovascular rehospitalizations were also observed in the intervention arm, suggesting that the multidomain rehabilitation may enhance overall resilience and recovery beyond cardiac mechanisms. In prespecified subgroup analyses (not adjusted for multiple comparisons), effects were consistent across age, sex, race, and multiple comorbid conditions, underscoring the intervention’s efficacy and generalizability.^12, 17–19^ The notable exception was heart failure phenotype.^14^

Participants with acute heart failure with preserved ejection fraction (HFpEF)—who comprised 53% of the phase 2 cohort—had significantly worse baseline impairments yet derived ~50% larger improvements across physical and psychosocial outcomes, along with more favorable trends in mortality, rehospitalization, and healthcare costs, compared to those with reduced ejection fraction (HFrEF).^10,14,20^ These findings identify acute HFpEF as a uniquely vulnerable yet highly modifiable phenotype and provide strong scientific premise for a definitive, event-powered trial.

HFpEF is the most common and fastest growing HF phenotype among older adults, disproportionally affecting women and non-Hispanic Black individuals.^21–23^ It is increasingly recognized as a multisystem geriatric syndrome driven by aging, inflammation, physical inactivity, and multimorbidity—factors that contribute to physical dysfunction, high frailty burden, poor HRQOL, and rising mortality.^22–24^ Despite its substantial burden, patients with HFpEF and recent hospitalization have been systematically excluded from most exercise-based trials and are ineligible for cardiac rehabilitation.^16,24–26^ As a result, rigorous and generalizable clinical outcomes data are urgently needed to inform clinical decision-making and guide rehabilitation policy for this high-risk group.

To address these gaps, we were funded by the National Institute of Aging (NIA) to conduct *Physical Rehabilitation for Older Patients with Acute HFpEF (REHAB-HFpEF)*, a phase 3, multicenter, randomized controlled trial of the multidomain REHAB-HF intervention. We hypothesize that the intervention will reduce the primary endpoint of combined all-cause rehospitalizations and mortality and improve major mobility disability (MMD) and HRQOL at 6-month follow-up, during the high-risk post-ADHF period^27^ (Figure 1). This trial directly addresses national priorities articulated by the 2019 NIA workshop on HFpEF^24^ and the recent American Heart Association/American College of Cardiology scientific statement on exercise training in HFpEF,^28^ and, if successful, will provide the critical evidence needed to inform clinical guidelines, support CMS coverage decisions, and establish a scalable rehabilitation pathway for older adults with acute HFpEF.

## Methods

### Study design and setting

**Figure 2.**
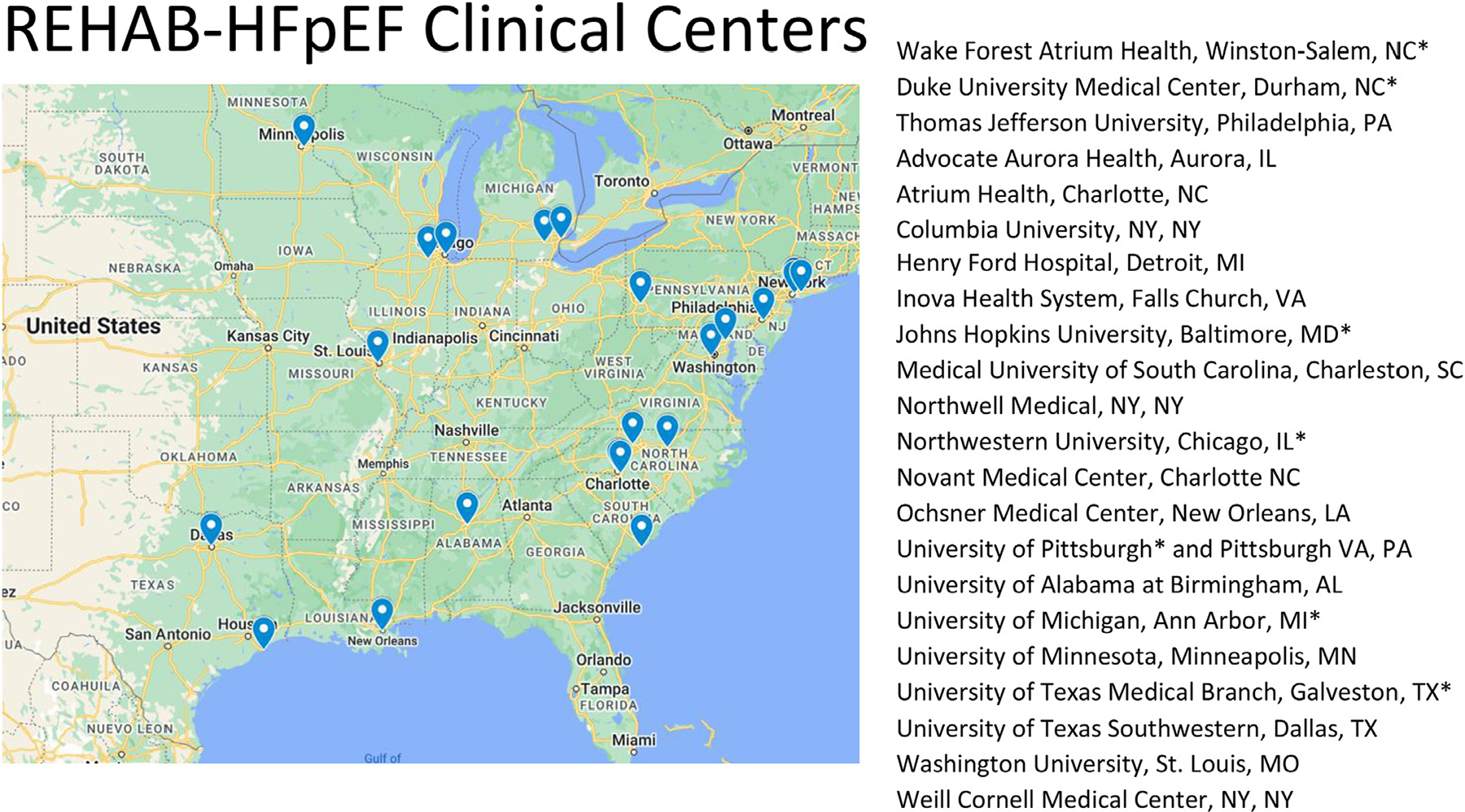
REHAB-HFpEF clinical centers. REHAB-HFpEF clinical centers are geographically distributed across multiple U.S. regions. Sites were selected for their strong track record in enrolling and supporting a broadly representative population of older adults with ADHF and HFpEF. *Indicates sites affiliated with a Claude D. Pepper Older Americans Independence Center.

REHAB-HFpEF is a multicenter, randomized, single-blind, attention-controlled phase 3 trial aiming to enroll 880 older patients (≥60 years) with HFpEF hospitalized with ADHF. Leveraging the “hub-and-satellite” model from REHAB-HF and its predecessor, HF-ACTION,^25^ participants will be recruited across 22 U.S. health system clinical centers (hubs), each potentially linked to up to three affiliated community hospitals (satellites) to ensure broad representation (Figure 2, Appendix).

### Study population

Inclusion and exclusion criteria are largely based on the REHAB-HF phase 2 trial,^9,12,29,30^ but restricted to patients who appeared most responsive–those with acute HFpEF (EF ≥45%). This EF threshold matches phase 2 and facilitates implementation. Participants are screened and enrolled during the index ADHF hospitalization. ADHF is confirmed by a study physician using a standard definition: ≥1 HF symptoms, ≥2 HF signs, and a change in HF-specific medical therapy (Table 1). Eligible patients are ≥60 years, able to ambulate ≥4 m independently (with or without a gait assistive device), perform basic activities of daily living independently prior to admission, and be expected to return home at discharge.

Key exclusion criteria are acute myocardial infarction; severe valvular disease; infiltrative cardiomyopathy; advanced kidney disease (estimated glomerular filtration rate ≤20 mL/min/1.73 m^2^ or dialysis); regular moderate to vigorous exercise before hospitalization; or other conditions limiting safe participation. Cognitive impairment, common in older adults hospitalized with HF,^29,31^ is assessed using the Montreal Cognitive Assessment.^32^ Patients with scores 10 to 17 are eligible for enrollment if adequate social support for adherence is confirmed by the study team.

To ensure safety and consistency across sites, eligibility is confirmed using a standardized screening process combining medical record review and clinical assessment by the site investigator in consultation with the treating team. This includes evaluation of medical stability, anticipated recovery trajectory, and ability to safely participate. Patients with noncardiovascular conditions expected to limit 1-year survival are excluded. This structured approach, used successfully in phase 2, allows clinical judgment while minimizing variability across sites. Intervention delivery is led by physical therapists experienced in geriatric multimorbidity, supporting safety throughout the trial. Oversight is provided by an NIH-appointed Data Safety Monitoring Board (DSMB) (see Appendix for trial organization).

Patients who provide informed consent and meet eligibility criteria are randomized 1:1 to the REHAB-HF intervention or attention control arm using permuted block randomization with stratification by clinical center.

The trial is conducted in accordance with the Declaration of Helsinki and was approved by the central Institutional Review Board at Wake Forest University Health Sciences, with reliance agreements from all participating institutions. The trial is registered with Clinicaltrials.gov (NCT05525663).

### Usual care

Participants in both arms receive clinician-directed usual care, which may include physical therapy or occupational therapy (hospital, outpatient, and/or at home-based), and cardiac or pulmonary rehabilitation during follow-up. Disease management, including medications, device therapy, and HF management, remains entirely at the discretion of treating clinicians and are not altered by the study protocol for either arm. Any clinical concerns raised by participants or identified by study staff are referred to their clinicians. Providing full access to usual care in both arms, as in phase 2, was an intentional strategy to minimize differential exposure to nonstudy therapeutic services.

### Attention control arm

To minimize differential study staff contact, attention control participants receive structured telephone and in-person contact during the 12 months following the index hospitalization, mirroring contact frequency in the intervention arm (Table 2 and Supplementary Figure 1). Study staff conduct biweekly telephone calls at weeks 2, 4, 6, 8, and 10, followed by monthly calls at months 4, 5, and 12. Participants attend in-person reassessments at months 3 and 6 for evaluation of physical function and HRQOL. At each contact, staff collect information on symptoms, HF disease management programs, medical adherence, physical activity, rehabilitation received, healthcare utilization, HRQOL, and clinical events. Participants are encouraged to follow all usual care recommendations, but do not receive any specific rehabilitation recommendations or exercise prescription from study personnel.

### REHAB-HF intervention arm

#### Overview:

Details of the REHAB-HF intervention have been previously published.^12,30,33^ In brief, this multidomain physical rehabilitation intervention, developed in phase 2, is tailored for older adults with HFpEF hospitalized for ADHF, a population with heterogeneous mobility, multiple comorbidities, and high frailty.^10, 34–37^ The intervention targets physical function deficits, worsened by acute illness and hospital-associated immobility, with an initial emphasis on regaining strength, balance, and functional mobility to allow safe participation in walking-based endurance activity. The goal is to improve performance across four physical function domains essential for functional independence—strength, balance, mobility, and endurance—by utilizing standardized, targeted exercises with specific progressive milestones to prevent mobility disability. The intervention begins during hospitalization and extends through outpatient and home-built environment settings, providing a transitional dose of rehabilitation from stabilization after acute decompensation through recovery and back to chronic disease state.

Inpatient sessions occur once daily for ~45 minutes and focus on balance, mobility, and functional strength, using exercises that can easily be administered in a hospital room with minimal equipment. Then, within 7 days after hospital discharge, 60-minute outpatient sessions are conducted 3 days/wk for a target total of 36 sessions. One-on-one (participant to interventionist), inperson delivery is the preferred mode due to the fall risk, frailty, multimorbidity, and cognitive dysfunction prevalent in this population, all of which require close supervision and frequent adaptation. For very debilitated participants, home-based sessions may be provided until they can safely attend outpatient sessions.

Outpatient sessions are complemented by independent home exercise, including strengthening exercises and low-intensity walking on nonfacility days, progressing toward 30 minutes. Home exercise begins after an extensive home-built environment assessment visit,^38^ which identifies safe and accessible areas for exercise, and highlights salient social determinants of health that the team can address to promote intervention adherence (ie, transportation needs, support person engagement).^33^

If outpatient sessions are interrupted by rehospitalization or illness, inpatient intervention sessions resume once the participant is medically stable. The study team also maintains close contact with the participant and their providers to support retention and ensure safe, timely resumption of outpatient sessions or home sessions (as a bridge back to outpatient) following hospital discharge.

**Figure 3.**
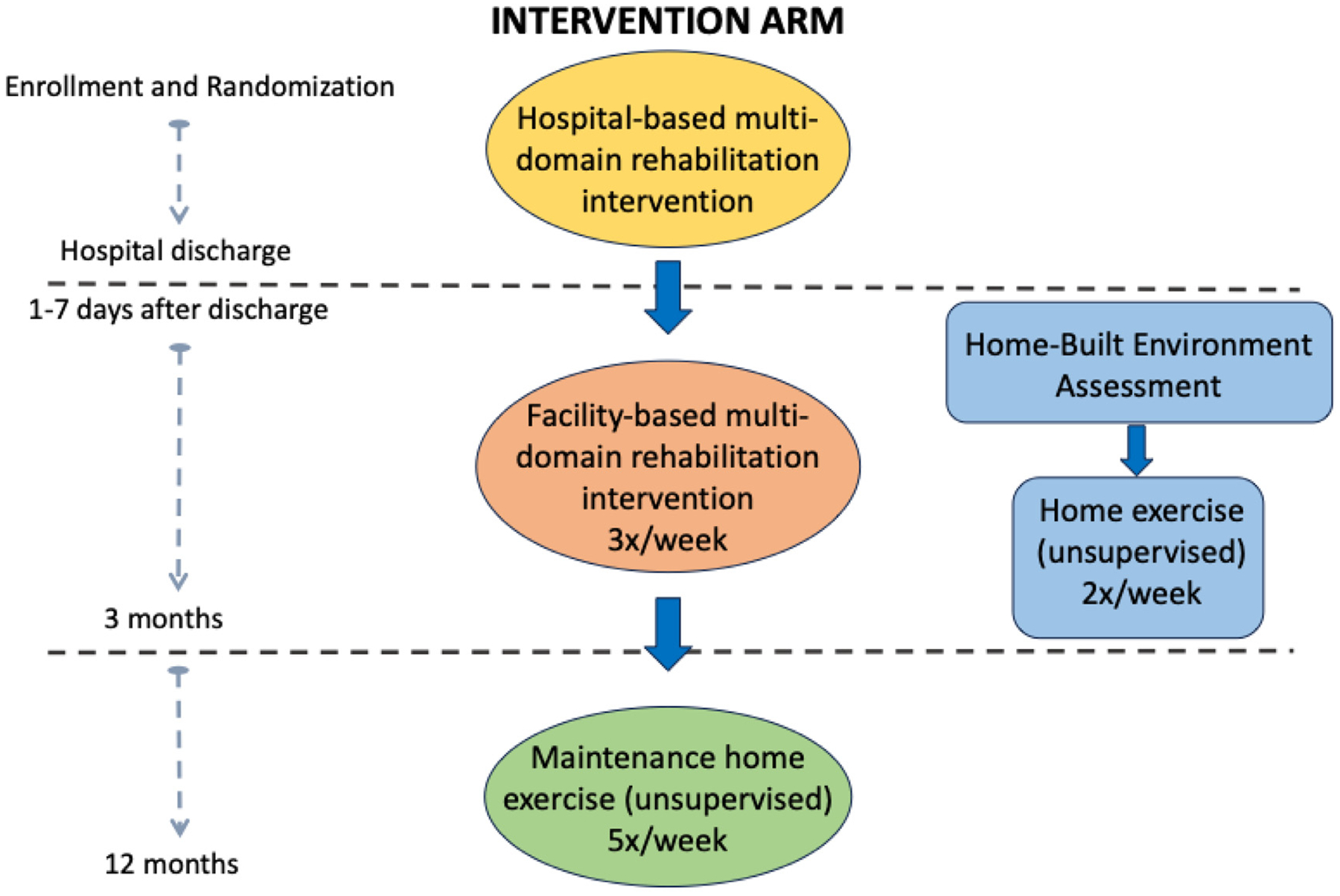
Flow of events in the REHAB-HFpEF intervention arm. Outlined are the timeline and structure of the intervention arm for participants enrolled and randomized in the REHAB-HFpEF trial. **• Inpatient-based phase (purple):** The multidomain rehabilitation intervention begins during the index hospitalization. **• Facility-based phase (yellow):** Participants attend outpatient, facility-based multidomain rehabilitation sessions, beginning ideally within 7 days of hospital discharge and continuing three times per week for 3 months. **• Home-built environment assessment (blue):** Conducted 1 to 7 days postdischarge, this assessment informs tailoring of the home exercise component. **• Home exercise component (blue):** Participants complete unsupervised home exercise sessions twice weekly in parallel with facility-based sessions. **• Maintenance phase (green):** Beginning in month 4 postdischarge, participants transition to fully unsupervised home exercise five times a week. Arrows indicate the sequence and progression through the phases, ensuring a structured and scalable rehabilitation approach designed to improve functional status, quality of life, and clinical outcomes.

A key goal, which is addressed early and throughout the first 3 months, is preparing the participant for the independent, unsupervised home maintenance phase beginning at month 4. Near the 3-month visit, participants receive an individualized maintenance exercise prescription and recommendations for community-based exercise resources. This schedule achieved excellent retention and adherence in phase 2.^12,17^ Flow of the intervention arm is presented in Figure 3.

#### Exercise prescription:

The broad range of exercises included in the study are designed to accommodate the heterogeneous capabilities of participants. Exercises are individually tailored based on functional performance (1–4, from lowest to highest) for each domain using objective criteria (Table 3). Exercises are then selected to match the participant’s functional level; sample exercises are shown in Table 4. Based on phase 2, where 14% of participants were initially at level 1 for most domains, 36% were at level 2, and 29% were at level 3,^12^ we anticipate a broad range of baseline levels in this phase 3 trial.

**Figure 4.**
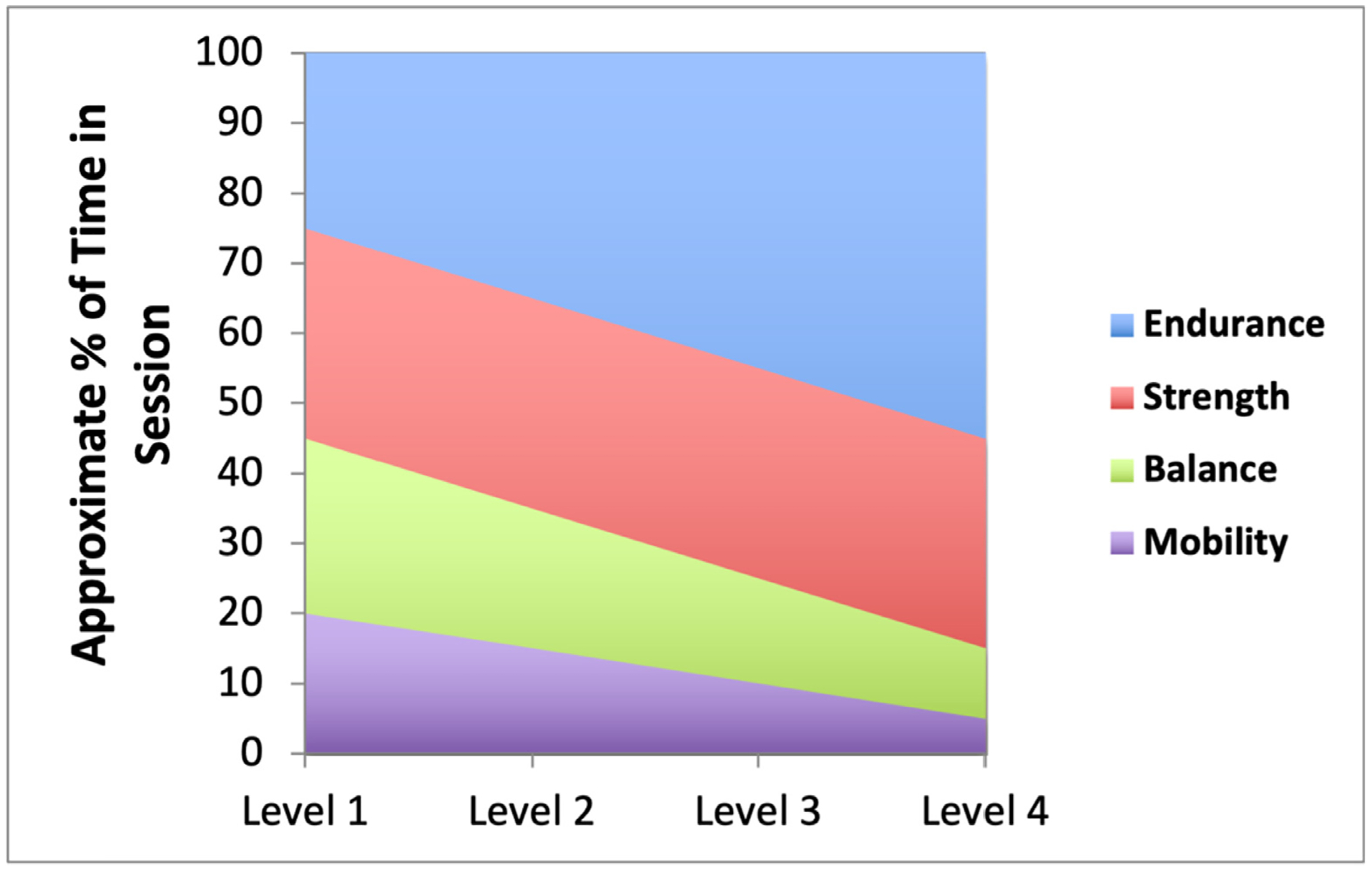
Percent of time in session by functional domain and performance level. The distribution of time spent across exercise domain during intervention sessions is stratified by functional performance level (level 1 = lowest, level 4 = highest). Time allocation is tailored to each participant’s needs using the REAHB-HF stratification grid. For example, a participant with substantial balance and mobility impairments will initially spend more time on those domains, with less time on endurance. As performance improves, balance and mobility receive less emphasis, and endurance training increases. Conversely, participants with modest baseline impairments may focus more on strength and endurance from the outset. While individualized, sessions generally follow a progression toward greater emphasis on endurance training as strength, balance, and mobility improve. Adapted and reprinted from ref. [30], ©2017 with permission from Elsevier.

Exercise progression over time is individualized based on performance level within each domain, with the relative proportion of session time per domain tailored to the pattern of impairments (Figure 4). Rate of perceived exertion guides intensity and progression. Participants are continually challenged to improve gradually in small increments from session to session. A key goal is to increase endurance (walking time); doing this safely requires first addressing deficits in balance, strength, and mobility, as standard endurance training without first addressing these deficits has reduced efficacy and increased injury risk in frail patients.^39–41^

The specific exercises for each domain have been described previously in detail.^30,33^ In brief, functional strength exercises of the lower extremities are supplemented by general resistance exercises for major muscle groups of the upper and lower extremities. Balance exercises include both static and dynamic activities. Mobility exercises combine mobility and balance activities, such as dynamic start and stop, and changing direction while walking. Close supervision and guarding are provided to prevent injuries and falls. Endurance training begins with repeated bouts of usual-pace ambulation with rest breaks as needed, progressing from 5 to 10 minutes total toward sustained walking at target rate of perceived exertion up to 40 minutes (level 4). Walking is the preferred endurance mode, but other modalities (ie, stationary up-right or recumbent cycling or 4-extremity recumbent or arm ergometry) may supplement when needed.

#### Safety:

Prespecified safety protocols based on vital signs (heart rate, blood pressure, pulse oximetry; telemetry not included) and reported symptoms are followed as detailed in phase 2.^30,33^ For participants with insulin-dependent diabetes mellitus, blood glucose is monitored before and after exercise sessions for at least the first six sessions to establish patterns, and as needed thereafter.

#### Intervention fidelity:

**Figure 5.**
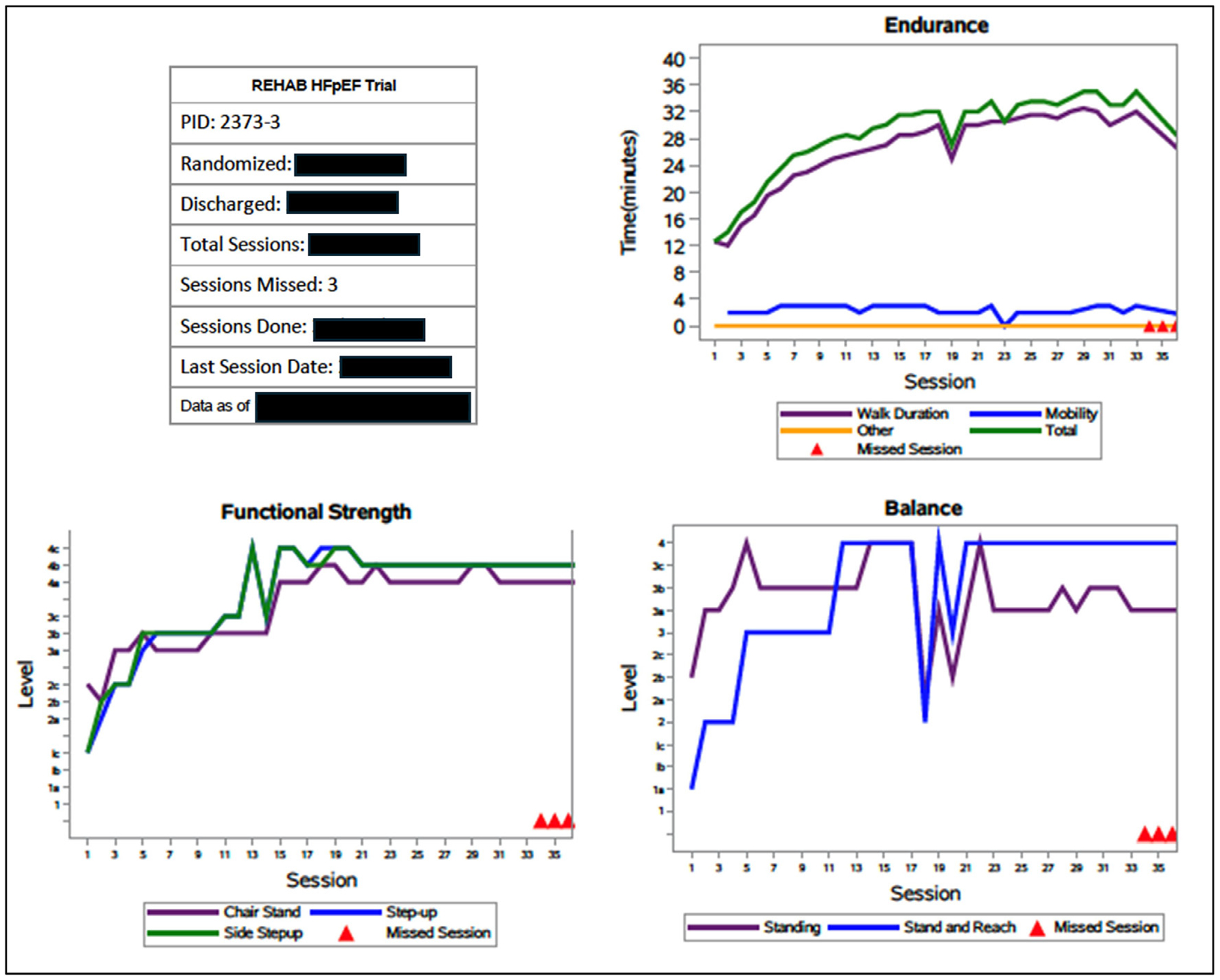
Participant progress report. The participant progress report (PPR) displays attendance and domain-specific progress for a participant actively engaged in the intervention arm. The endurance graph (upper right) tracks time spent on walking, other endurance activities (eg, stationary bicycle, 4-extremity recumbent cycle), and mobility exercise. The total time (green line) represents the combined duration of all endurance activities. The functional strength graph (lower left) shows progression in sit-to-stand, forward step-up, and side step-up exercises. The balance graph (lower right) illustrates progression in static standing balance and dynamic stand-and-reach beyond base of support; midintervention spikes reflect lower-level exercises performed with eyes closed. In this example, the participant attended 33 of 36 scheduled sessions (92% attendance). Overall, progress is evident across all domains, with increased time and higher functional levels achieved over the course of the intervention. Notably, improvements in functional strength and balance correspond with enhanced endurance capacity and sustained walking time.

Intervention fidelity strategies, based on the NIH Behavior Change Consortium Treatment Fidelity Workgroup recommendations and used successfully in phase 2, have been previously described in detail.^17,30,33^ Briefly, the intervention follows a standardized protocol with defined exercises across four domains, with selection and progression guided by standardized assessments (Table 3) and detailed incremental progressions. Interventionists receive centralized virtual and in-person training, including protocol review, demonstration videos, and hands-on practice, with annual refresher sessions to prevent protocol drift. Fidelity is monitored continuously through documentation of exercises performed at each session, and participant progress tracked in a study-wide database. Participant progress reports (PPRs, Figure 5) are reviewed during biweekly video conference meetings of the Sustaining Participant Engagement Committee (SPEC), which includes trial leadership and site intervention and clinical coordinator leads. (See Appendix for trial organization.) Site leads then review PPRs with treating interventionists or ancillary coordinators, relay trial leadership recommendations, and address any challenges. Interventionists also review PPRs with participants. Any concerns regarding protocol implementation are promptly addressed. Extensions to the intervention period due to intercurrent illness or rehospitalization, as permitted in the protocol, are reviewed and approved by SPEC leadership consensus.

#### Interventionist characteristics and collaboration:

Each site has a Site Intervention Leader, a senior physical therapist who oversees the intervention and supervises the interventionists. Interventionists are physical therapists, preferably with cardiovascular and pulmonary or geriatric board certification, or exercise physiologists or physical therapy assistants working in collaboration with physical therapists. All interventionists are experienced in working with frail older adults who have multiple chronic conditions and a wide range of fitness and functional abilities.

At least two interventionists per site are trained for inpatient and outpatient/home-based delivery to minimize gaps in care (eg, holidays, sick days) and to limit individual interventionist effects. In outpatient settings, physical therapists may collaborate with physical therapy assistants and exercise physiologists, depending on participant functional level (Table 3). Physical therapists oversee the intervention, perform the home-built environment assessment, deliver all inpatient and home-based sessions, and provide all outpatient sessions for lower functioning participants (functional level 1 and 2, shown in red in Table 3). For higher-functioning participants (functional level 2 or 3 in the yellow domains, or functional level 4 in green), handoffs to assistants or exercise physiologists may occur under physical therapist oversight. Following any rehospitalizations or intercurrent illnesses, the physical therapist reassesses functional level and re-establishes the exercise prescription.

### Retention and adherence

Retention and adherence strategies, grounded in the National Institutes of Health Behavior Change Consortium Treatment Fidelity Workgroup recommendations, begin at enrollment and continue throughout the trial. Detailed descriptions of these fidelity and safety procedures have been published previously in.^17,30,33^ Key elements include:

- Early identification and mitigation of modifiable medical and social barriers (eg, transportation, competing appointments),
- Engagement of care partners and social supports,
- Clear communication of expectations,
- Procedures for managing interruptions (eg, rehospitalization), and
- Ongoing monitoring of participant progress to allow early action when adherence wanes.

The SPEC meets biweekly to review the participant progress, monitor adherence, address barriers to retention, and ensure timely outcome ascertainment.

These strategies proved effective in phase 2, which achieved a 78% intervention session attendance rate after adjusting for sessions missed due to medical illness. Attendance was independently associated with improvements in physical function, HRQOL, depressive symptoms, as well as a 3% reduction in the combined risk of all-cause rehospitalization and death per session attended, demonstrating a dose-response relationship. Retention was 82% for the primary functional outcome (Short Physical Performance Battery, SPPB) and 99% for all-cause rehospitalization and death, outcomes now central to this phase 3 trial.

Adherence process measures for the trial include the percent of sessions attended (raw and medically adjusted), number and reasons for missed sessions, exercises performed in each domain at all intervention sessions, phone contacts and study visits completed, and outcomes ascertained.

Limited extensions (2–4 weeks) are allowed for both study arms to accommodate illness or rehospitalization and support completion of the intervention and outcomes assessments.

### Pragmatic elements

We intentionally designed REHAB-HFpEF to incorporate multiple pragmatic features while maintaining rigor required for a definitive efficacy trial, thereby positioning any positive findings for rapid clinical uptake. Pragmatism was formally evaluated using the PRECIS-2 framework, which scores nine trial design domains from 1 (very explanatory) to 5 (very pragmatic).^42^ The trial scored 36/45 (mean score of 4 per domain), indicating a “rather pragmatic” hybrid design (Table 5; Supplementary Figure 2). Lower scores for Flexibility: Adherence and Flexibility: Follow-up reflect structure for robust efficacy assessment. Overall, this profile enhances scalability, supports dissemination across diverse clinical environments, and strengthens real-world generalizability.

### Assessments and outcomes

Clinical events are collected at each visit and through interim phone calls with participants, family, and caregivers, supplemented by the EHR and the National Death Index. Rehospitalizations (≥24-hour stays for any cause; categorized per Horwtiz et al^43^) are tracked along with deaths, emergency department/observation visits (< 24 hours), unscheduled or urgent medical visits (including same-day HF clinic visits), nursing home placement, falls, and nonstudy rehabilitation (eg, outpatient and home health physical therapy and/or occupational therapy, cardiac or pulmonary rehabilitation). Events are categorized by cause as HF-related, other cardiovascular, or noncardiovascular and evaluated for potential relationship to the intervention. Final determinations are made by an independent, blinded Events Adjudication Core (see Appendix for trial organization) using an adapted protocol from SPRINT.^44^

The primary outcome is the combined count of all-cause rehospitalizations and death at 6 months. This composite was selected because rehospitalizations are common after ADHF and have substantial impact on health trajectory, HRQOL, and functional status, outcomes of critical importance to this population.^45^ The all-cause composite endpoint captures the full patient experience, recognizing that adverse events in this population are often driven by frailty, multimorbidity, and multiorgan dysfunction, factors plausibly modifiable by the multidomain rehabilitation intervention, which likely exerts pleiotropic effects.^28,46,47^ Phase 2 results suggest that the intervention may improve resilience and reduce clinical events by targeting physical frailty.^48^ Use of the adjudicated, all-cause composite reduces bias, and analyzing the total number of events, rather than time to first event, increases statistical power and better reflects overall disease burden, as demonstrated by phase 2 data showing an average 1.50 (intervention) to 1.61 (control) rehospitalizations per patient over 6 months. These outcomes are widely used in definitive trials aiming to influence clinical guidelines and national coverage decisions, essential for dissemination and implementation.^16^

Key secondary outcomes include prevalence of MMD, defined as inability to walk 160 m in the 6-minute walk test (6MWT), and disease-specific HRQOL measured by the KCCQ at 6 months. Together, MMD and KCCQ capture objective physical performance and patient-perceived health status, providing a comprehensive assessment of recovery.

MMD was selected for its clinical relevance and methodological precedent. It is highly valued by older adults, prevalent in the target population, associated with adverse events and reduced HRQOL,^49^ and was responsive to the REHAB-HF intervention in the phase 2 trial. The 6MWT was chosen over the 400-meter walk test used in prior NIA-sponsored trials such as LIFE^50^ because of feasibility constraints in frail, hospitalized populations and limited walking space in inpatient and home settings. The LIFE study’s walking speed threshold (~0.44 m/s) aligns with 6MWT-based thresholds (~0.4 m/s) associated with limited community ambulation^51^ and predictive of adverse outcomes.^49,52,53^ Assessment at 6 months enables evaluation of the intervention’s durability during the self-directed Maintenance Phase. If a participant cannot perform the 6MWT due to logistical barriers (eg, inability to travel to the clinic or lack of suitable space), MMD will be adjudicated using alternative criteria: inability to walk 4 m in ≤10 seconds or documentation of inability to walk across a room based on participant, proxy, or medical record report. Participants meeting these criteria are considered functionally unable to complete 160 m.

The KCCQ^54^ was selected as a validated, disease-specific measure that quantifies symptom burden and physical and social limitations. It has strong prognostic value, independently predicting hospitalization and mortality across the HF disease spectrum, including ADHF. In phase 2, baseline scores were markedly low (~41), and the intervention produced clinically meaningful improvements at 3 months (between-group differences +6.9), exceeding the 5-point threshold for significance.^55^ Improvements were consistent across all subscales and partially mediated by physical function gains, underscoring the KCCQ’s sensitivity to both functional and broader patient-perceived benefits.

Six-month follow-up was selected for the primary and secondary outcomes as this is the highest-risk period after ADHF hospitalization, with risk returning to prehospitalization levels thereafter.^27^ To assess intervention durability, clinical events are collected for up to 12 months after randomization, or as long as feasible for participants enrolled late in the trial, with ~90% excepted to have 12-month follow-up data.

As shown in Table 2, additional assessments include medication use, medical history, healthcare resource utilization, and blood samples with plasma and serum aliquots banked for future analyses. Functional status is evaluated using objective measures: 6-minute walk distance,^56–59^ SPPB,^60^ modified Fried frailty status^61^ and Montreal Cognitive Assessment.^32^ Additional patient-reported outcomes include the 12-Item Short-Form Health Survey (SF-12),^62–64^ the EuroQol (EQ-)5D-5L,^65^ and the 15-item Geriatric Depression Scale (GDS-15).^66,67^ Also, a novel deficit accumulation frailty index developed by our team will be used to explore predictors of intervention response.^68–71^

### Statistical considerations

#### Sample size

Based on phase 2 data, the 6-month combined all-cause rehospitalization and death rate in the control group is conservatively estimated at 1.46. Enrolling 880 participants (~40 per center) provides 90% power (two-sided, alpha = 0.05) to detect an 18% reduction in the primary outcome, an effect size consistent with other major lifestyle and rehabilitation trials such as the Look AHEAD (18%) and LIFE (20%).^50,72^ These projections allow for 5% loss to follow-up and align with statistical methods planned for the primary outcome analysis. The targeted sample size also provides > 90% power to detect an absolute between-group difference as small as 11% in the prevalence of severe MMD at 6 months. Phase 2 results indicate that the average between-group difference in KCCQ at 3 months was ~6 points. With a pooled standard deviation of 25.2 and a conservative estimate of 25% evaluable participants for KCCQ, the trial retains > 90% power to detect this difference. The sample size further provides adequate power to detect relevant intergroup differences and correlations for other key outcomes.

#### Statistical analysis plan

All analyses will follow the intention-to-treat principle. Count data (eg, number of combined all-cause hospitalizations and deaths, total hospitalization days) will be analyzed using Poisson or negative binomial regression models, with the natural log of the follow-up time as an offset. Longitudinal continuous outcomes (eg, KCCQ scores) will be analyzed using linear mixed effects models to estimate the intervention effects. Longitudinal binary outcomes (eg, prevalence of MMD) will be analyzed using generalized linear models, including generalized estimating equations for repeated measures. Contrasts will be used to estimate intervention effects at months 3 and 6. Time to first postrandomization occurrence of an event will be evaluated using survival analyses. Analyses will adjust for appropriate covariates, including geographic region of the clinical center, age, and sex. Both primary and secondary outcomes will also undergo supplemental baseline-adjusted analyses using baseline variables that are significantly prognostic of the outcomes, selected by an objective regression approach consistent with methods used in HF-ACTION.^25^

In addition to the primary analyses, two prespecified supporting analyses will leverage data from both the completed phase 2 REHAB-HF trial and the phase 3 REHAB-HFpEF trial, which utilized similar study designs, an identical intervention protocol, and harmonized data collection procedures for rehospitalization and mortality outcomes. First, a pooled participant-level analysis combining data from this phase 3 trial and data from the HF-pEF participants in the phase 2 trial will be conducted to increase precision of effect estimates and evaluate consistency of treatment effects across trial phases. Second, a Bayesian borrowing analysis will be performed, incorporating phase 2 results as informative priors to quantify the posterior probability of clinically meaningful benefit in phase 3.

Prespecified subgroup analyses will assess whether intervention effects vary by characteristics hypothesized to influence responsiveness to rehabilitation: sex; race and ethnicity; age; overweight or obesity; NYHA Class; atrial fibrillation; diabetes; ischemic heart disease; chronic obstructive pulmonary disease; chronic kidney disease; depressive symptoms; cognitive impairment; and frailty. Sensitivity analyses will examine the effect of missing data on primary outcome inference. A detailed Statistical Analysis Plan will be finalized before database lock and will delineate all primary and secondary analytical procedures for the trial.

The NIA-appointed Data Safety Monitoring Board (DSMB) (see Appendix for trial organization) will monitor participant safety and overall study conduct. No interim analyses for efficacy or futility are planned.

### Economic analysis

Incorporating an economic analysis will help inform coverage policy and facilitate implementation if the trial is positive. We will evaluate: (1) medical resource use, costs, and preference-based HRQOL; and (2) the long-term cost-effectiveness of the rehabilitation intervention vs attention control. To inform these analyses, we are collecting data on: medical resource use; intervention resources; patient time; and health status using the 5-level EQ-5D-5L. Costs will be valued from both the health-care system and societal perspectives, with the latter inclusive of indirect costs of patient time associated with the intervention.^73^ Medical resource cost will be valued using published national average Medicare payment rates. Intervention-associated costs will be estimated using the TEAM-HF Costing Tool^74^ that we designed to quantify costs associated with patient-centered interventions. Professional and patient time for the rehabilitation sessions and travel will be valued using national average wage rates from the U.S. Bureau of Labor Statistics. Utility weights will be mapped from EQ-5D-5L responses using Pickard et al^75^ value set. Generalized linear models will be used to compare mean resource use, costs, EQ-5D-5L utility weights, and estimated quality-adjusted life-years over the 6-month follow-up period between the rehabilitation and attention control arms. To evaluate the expected long-term cost-effectiveness of the rehabilitation intervention, we will apply the TEAM-HF Cost-Effectiveness Analysis model that we designed to estimate long-term costs, survival, and quality-adjusted survival for patients with HF.^76^ The TEAM-HF model relies on the Seattle Heart Failure Model (SHFM) to generate estimates of projected survival based on patient demographic characteristics and intervention effects on medications, clinical and laboratory measures, and HRQOL. To convey uncertainty associated with modeled estimates, we will conduct probabilistic sensitivity analyses and report the percentage of simulations with estimated incremental cost-effectiveness ratios below a threshold of $100,000 per quality-adjusted life-year gained.^77^

## Discussion

REHAB-HFpEF is a phase 3, multicenter, single-blinded, attention-controlled randomized clinical trial designed to definitively evaluate whether a transitional, tailored, structured, and progressive multidomain physical rehabilitation intervention can reduce 6-month combined all-cause rehospitalizations and mortality and improve MMD and HRQOL in older adults hospitalized with acute HFpEF. As the largest exercise-based trial in this population to date, REHAB-HFpEF directly addresses a long-standing absence of an evidence-based rehabilitation pathway for a clinically vulnerable group with substantial functional limitations (Supplementary Figure 3).

Current CMS coverage for cardiac rehabilitation, established following HF-ACTION,^25^ applies only to clinically stable HFrEF (LVEF ≤35%) patients who remain symptomatic despite ≥6 weeks of optimal medical therapy and have not experienced a recent cardiovascular hospitalization. In contrast, REHAB-HFpEF intentionally targets older adults medically stabilized for discharge following acute HFpEF (LVEF ≥45%) hospitalization. Thus, REHAB-HFpEF aims to extend the HF-ACTION paradigm by generating the first event-driven evidence for a multidomain physical rehabilitation intervention in acute HFpEF. Notably, CMS has cited the absence of definitive clinical outcomes as a key barrier to approval of a rehabilitation pathway for this population.^16^ In addition to the coverage restriction, standard cardiac rehabilitation approaches, which emphasize conventional aerobic and resistance training, are poorly suited to the substantial multisystem impairments and multidomain deficits—including mobility and balance limitations—and the high frailty burden characteristic of acute HFpEF and may pose safety concerns.^4,15,16,29,78^ These limitations underscore the need for a therapeutic strategy aligned with the specific physiological and functional vulnerabilities of this population.

The REHAB-HF multidomain intervention was developed to directly address the complex, multidomain pattern of impairments observed in older adults with acute HFpEF. It incorporates structured progression rules and individualized exercise prescriptions across balance, mobility, strength, and endurance domains.^12^ Transitional delivery across inpatient, outpatient, and home-built environment settings facilitates continuity through the period of highest vulnerability post-ADHF hospitalization.^27^ These design features are grounded in the successful phase 2 REHAB-HF framework and incorporate comprehensive fidelity procedures to ensure consistent, safe implementation across diverse sites, as detailed in our previously published intervention and fidelity manuscripts.^17,30,33^ Collectively, these elements support internal validity and the potential for future scalability.

REHAB-HFpEF also integrates multiple pragmatic design features intended to maximize generalizability and facilitate downstream implementation. Broad inclusion criteria, minimal exclusions, and recruitment during usual care hospital admissions allow enrollment of participants who reflect the real-world HFpEF population. Purposeful recruitment strategies and a hub-and-satellite model enable participation from both academic medical centers and affiliated community hospitals, capturing a wide range of demographic, geographic, socioeconomic, and clinical contexts.

Operational feasibility was central to the trial’s design. The intervention integrates into existing workflows, using available rehabilitation personnel, clinical spaces, and standard therapeutic CPT codes without requiring specialized equipment or burdensome training. Adherence is systematically tracked; however, participants are not withdrawn from the study on the basis of session attendance, reflecting routine clinical practice. Primary outcomes are captured through the electronic health record to minimize participant burden, and an intention-to-treat analytic approach ensures comprehensive data inclusion. These pragmatic features, combined with rigorous randomization and event-driven outcomes, position REHAB-HFpEF to generate evidence that is clinically meaningful and directly translatable to health systems, payers, and patients, strengthening the potential for broad dissemination should the intervention prove effective.

Although REHAB-HFpEF does not include a formal process evaluation, the trial incorporates implementation-focused elements—such as fidelity and adherence monitoring, documentation of intervention delivery, and site-level tracking of resources, staffing, and training—that will inform scalability and translation to real-world practice.

As of this writing, enrollment has reached 66% (578/880) across 22 centers, with strong representation of women (61%) and people of color (43%); completion is anticipated in 2027.

## Conclusion

REHAB-HFpEF is uniquely positioned to provide definitive evidence on the clinical effectiveness of multidomain physical rehabilitation for older adults hospitalized with acute HFpEF, a population for whom no dedicated rehabilitation pathway currently exits. By rigorously evaluating clinical outcomes, functional abilities, and HRQOL in a broadly representative cohort, the trial has the potential to inform clinical guidelines, support CMS reimbursement decisions, and reshape rehabilitation practice for this expanding, high-risk population. If effective, the intervention may also serve as a scalable model for transitional rehabilitation in other high-risk populations characterized by multimorbidity and functional vulnerability.

## Supplementary Material

MMC1

MMC2

Supplementary material associated with this article can be found, in the online version, at doi:10.1016/j.ahj.2026.107420.

## Figures and Tables

**Figure 1.**
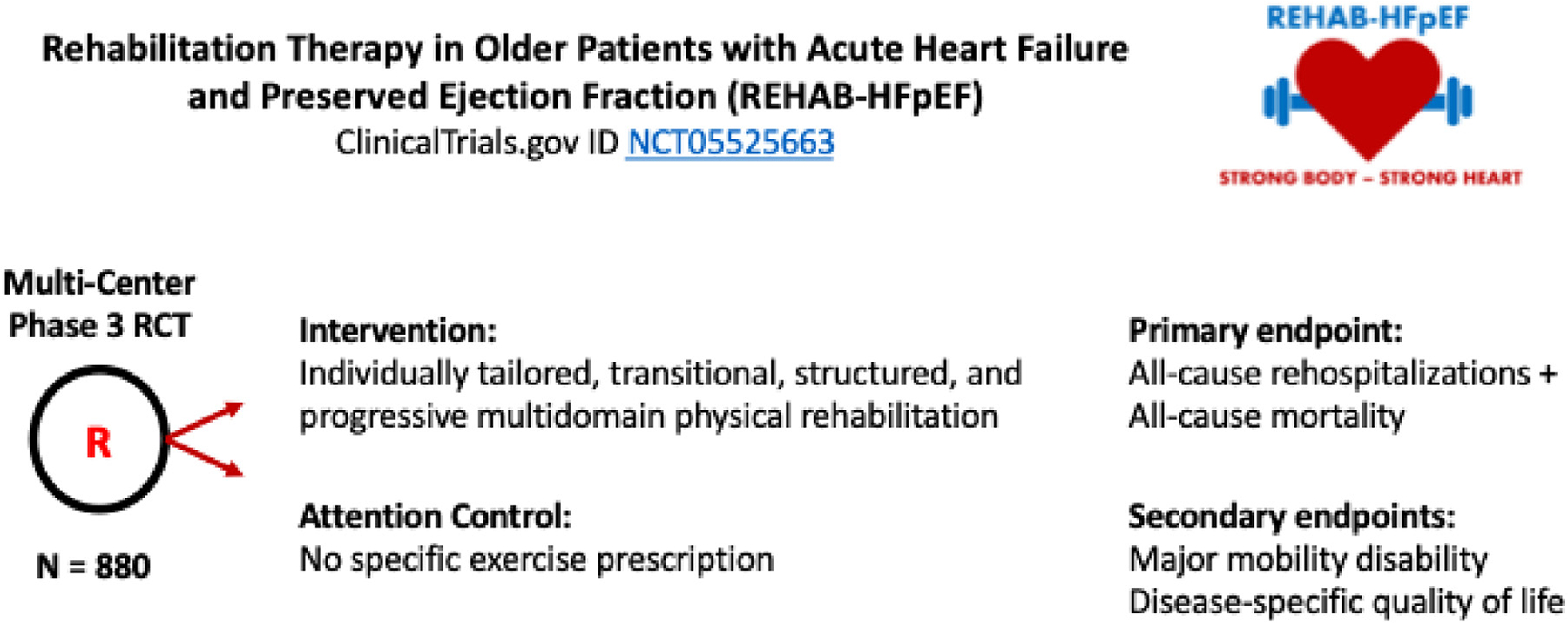
REHAB-HFpEF trial design. REHAB-HFpEF is a phase 3 randomized, single-blinded, attention-controlled trial enrolling 880 older adults hospitalized for acute decompensated heart failure (ADHF) with preserved ejection fraction (HFpEF). The trial tests whether a tailored multidomain physical rehabilitation intervention reduces all-cause hospitalizations and mortality (primary endpoint) as well as major mobility disability and disease-specific health-related quality of life (secondary endpoints) at 6-month follow-up. The intervention delivers progressive individualized training across domains essential for functional independence–strength, balance, mobility, and endurance–addressing a critical gap in recovery strategies for this high-risk, underserved population.

**Table 1.** REHAB-HFpEF inclusion and exclusion criteria

Inclusion criteria
Patients eligible for the trial must meet the following criteria at randomization:
Age ≥60 y old Ejection fraction ≥45% In the hospital setting > 24 h for the management of ADHF requires that all 4 of the following are met: At least one symptom of HF that has worsened from baseline: Dyspnea at rest or with exertion Exertional fatigue Orthopnea Paroxysmal nocturnal dyspnea (PND) At least two of the signs of HF Pulmonary congestion or edema on exam (rales) or by chest x-ray Elevated jugular venous pressure or central venous pressure ≥ 10 mm Hg Peripheral edema Wedge or left ventricular end diastolic pressure ≥ 15 mm Hg Rapid weight gain (≥5 lbs.) Increased b-type natriuretic peptide (BNP) (≥100 pg/mL) or N-terminal prohormone BNP (≥220 pg/mL) Change in medical treatment specifically targeting HF defined as change in dose or initiation of or augmentation of at least one of the following therapies Diuretics Vasodilators Other neurohormonal modulating agents, including angiotensin converting enzyme inhibitors, angiotensin II receptor blockers, beta-blockers, aldosterone, direct renin inhibitors, or sodium-glucose cotransporter-2 inhibitors Primary cause of symptoms and signs is judged by the site physician to be due to HF Clinical stability to allow participation in study assessments and the intervention Independent with basic activities of daily living prior to admission Ambulate 4 meters independently (with or without the use of an assistive device) at the time of enrollment
Exclusion criteria
At the time of randomization, none of the following conditions may exist:
Acute myocardial infarction based on clinical diagnosis within the past 3 mo Planned coronary artery intervention (percutaneous or surgical) within the next 6 mo Requiring care in an intensive care unit Severe aortic or mitral valve stenosis Severe valvular disease with planned intervention within the next 6 mo Known pericardial constriction, genetic hypertrophic cardiomyopathy, or infiltrative cardiomyopathy, including amyloid heart disease (amyloidosis) Advanced chronic kidney disease (eGFR < 20 mL/min/1.73 m^2^) or on dialysis or expected to be within the next 6 mo Terminal illness other than HF with life expectancy < 1 y Planned discharge other than to where the participant will live independently Impairment from stroke, injury, or other medical disorder that precludes participation in the intervention Known dementia by health record documentation, or MoCA ≤ 18, and without social support, or MoCA < 10, regardless of social support Already actively participating in regular moderate or vigorous exercise conditioning defines as >30 min per day, ≥twice per week consistently during the previous 6 wk Anticipated hospital discharge before baseline study measures could be completed

**Table 2.** Schedule of visits and assessments

	Index hospitalization (baseline)	Phone calls (wk 2, 4, 6, 8, 10)	3 mo visit	Phone calls (mo 4 and 5)	6 mo visit	12 mo phone call
Visit window	Prior to discharge	±3 d	90 ± 10 d*	120 and 150 ± 3 d	180 ± 10 d	365 ± 10 d
**Clinical events**	X	X	X	X	X	X
**Medications review**	X	X	X	X	X	X
**Medical resource use**	X	X	X	X	X	X
**6MWD**	X		X		X	
**SPPB**	X		X		X	
**Handgrip**	X		X		X	
**Frailty phenotype**	X		X		X	
**Biomarkers**	X		X			
**KCCQ**	X		X		X	
**SF-12**	X		X		X	
**EQ-5D-5L**	X		X		X	X
**GDS-15**	X		X		X	
**MoCA**	X		X		X	
**Geriatric conditions:**	X	X	X	X	X	X
**Falls, Urinary incontinence**						

*6MWD*, 6-minute walk distance; *EQ-5D-5L*, EuroQol 5-dimension 5-level; *GDS*, Geriatric Depression Scale; *KCCQ*, Kansas City Cardiomyopathy Questionnaire; *MoCA*, Montreal Cognitive Assessment; *SF*, Short Form; *SPPB*, Short Physical Performance Battery.

* May be extended due to circumstances (intercurrent illness, rehospitalization) that allow for extension of intervention.

**Table 3.** Exercise stratification grid: Performance levels for exercise prescription and collaboration overlay

	Level 1 (low function)	Level 2 (low-mod function)	Level 3 (mod function)	Level 4 (high function)
**Strength**: Lower extremity Sit to stand from a chair	Unable to rise from chair without UE	Able to rise from chair without using UE at least once	Able to rise from chair without UE 5x in > 15 sec but < 60 sec	Able to rise from chair without using UE 5x in ≤ 15 seconds
**Balance**: Standing	Unable to stand feet together 10 sec	Able to stand feet together 10 sec	Able to stand unsupported and reach forward 10’	Able to stand on 1 leg for 10 sec
**Mobility**: Walking speed over 4m (usual pace, assistive device permitted)	≤ 0.4 m/sec	> 0.4 m/sec but ≤ 0.6 m/sec	> 0.6 m/sec but ≤ 0.8 m/sec	Walk greater than 0.8 m/sec
**Endurance**: Continuous walking (usual pace; assistive device permitted)	Walk at usual pace for less than 2 min	Walk at usual pace ≥ 2 but < 10 min	Walk at usual pace for ≥ 10 but < 20 min	Walk at usual pace for ≥ 20 min

**Table 4.** Examples of exercise prescription by performance levels for each exercise domain

Exercise examples by domain	Level 1	Level 2	Level 3	Level 4
**Strength:**				
a) Sit to stand	On edge of chair leaning forward and pushing with hands	On edge of chair leaning forward with arms reaching out	In back of chair with arms across chest	As in level 3 at faster pace or from lower surface
b) Step-ups (front and side)	4-inch step*	6-inch step*	8-inch step*	10-inch step and/or with resistance*
**Balance:**				
Stand and reach	Stand with feet shoulder width apart; reach forward 6 inches and hold	Stand with feet shoulder width apart; reach forward 10 inches and hold	Stand with feet together; reach forward 6 inches (progressing to 10 inches) and hold	Semitandem stance; reach forward 6 inches (progressing to 10 inches) and hold
**Mobility:**				
Gait training	Stop and start abruptly	Brief accelerations during walking	Quick change of direction	Quick change of direction while engaged in activity requiring cognitive attention (eg, conversation, questioning)
**Endurance:**				
Continuous walking	Repeated brief bouts for a total duration of 10 min	Repeated brief bouts for a total duration of 20 min	Repeated bouts for a total duration of 30 min	Continuous for 20–30 min

Mode: Exercises appropriate to a participant’s performance level in each domain are selected as illustrated in the examples above.

Frequency and duration:

Inpatient: 30-minute sessions daily until discharge, with focus on domains to preserve functional mobility (typically 0–2 sessions). Outpatient 3 ×/week for approximately 60-minute sessions, integrating all domains (goal 36 sessions).

Intensity: Rate of perceived exertion (RPE) < 12 initially; increasing to 13 (11–15) for endurance; 15 to 16 for strength. Balance and mobility not to exceed endurance RPE.

Progression: As performance improves, participants advance to slightly more challenging exercises through structured, small increments. Performance is assessed during one-on-one training sessions, including standardized reassessment of functional performance in each domain.

* Performed with two, one, or no hands, holding on to support.

**Table 5.** PRECIS-2 pragmatic design evaluation of the REHAB-HFpEF study

Criteria	Score	Rationale
**Eligibility** (Who is selected to participate in the trial?)	4	Broad inclusion to include all older hospitalized HFpEF patients who are likely to be candidates for the intervention if it was being provided in a usual care setting Exclusion criteria minimal to enhance generalizability and enrollment: known contraindication to exercise, noncommunity dwelling, transferred to palliative care
**Recruitment** (How are participants recruited into the trial?)	4	Patients recruited during usual care encounters (hospital admission) Electronic Health Record algorithms established to identify all patients at hospital admission with acute HFpEF Potential participants present on their own accord (on HF admission)
**Setting** (Where is the trial being done?)	5	Diverse range of hospitals: ≥50% community-based, nonacademic Trial conducted in a setting identical to that which it is intended to be applied Intervention conducted in existing clinical/hospital settings or at home as needed
**Organization** (What expertise and resources are needed to deliver the intervention?)	4	Intervention can be delivered by existing rehabilitation personnel (eg, physical therapists, exercise physiologists) in usual care settings without intense training No special equipment needed Medical management left to usual care providers No usual care treatments excluded from intervention or control arms
**Flexibility: delivery** (How should the intervention be delivered?)	4	Intervention easily adaptable once started, including home delivery, reassessment of goals, and stratification of physical domains Includes a “toolbox” of options that can be selected to match the patient and setting Can be adjusted for rehospitalizations and intercurrent illnesses Flexible around usual care management
**Flexibility: adherence** (What measures are in place to make sure participants adhere to the intervention?)	2	Exercises inclusive of strength, balance, and endurance, tailored to the needs of the patient, and are progressed Feedback provided to patients Adherence monitoring and interventions provided to ensure adequate testing of intervention efficacy, which reduces this score Patients not withdrawn for poor adherence, which reduces this score
**Follow-up** (How are participants followed up?)	3	Primary outcome easily assessable via EHR Standardized secondary outcome assessments (major mobility disability) require patient for follow-up, may be more than usual care Follow-up period extends past intervention in order to assess long-term impact of intervention Maintenance phase is participant-driven
**Primary Outcome** (How relevant is it to the participants?)	5	All-cause rehospitalization and death are very relevant and important to the individual participants
**Primary Analysis** (To what extent are all data included?)	5	Intention-to-treat analysis Uses all available data

Total PRECIS-2 score: 36; average criteria score: 4.0.

## Key Points

- Largest physical rehabilitation trial in frail older adults with acute HFpEF.
- Tests a tailored multidomain program across hospital, outpatient, and home settings.
- Primary endpoint: 6-month all-cause re-hospitalizations and mortality.
- Powered secondary endpoints: major mobility disability and HRQOL.
- Designed to inform HFpEF care guidelines and coverage policies.
