# Supplementary material for: Physical rehabilitation for older patients with acute HFpEF (REHAB-HFpEF) trial: Design and rationale
Source: Am Heart J. Author manuscript; Available in PMC 2026 Apr 15. (PMC13077654; doi:10.1016/j.ahj.2026.107420)
Supplement: MMC1 [file NIHMS2162310-supplement-MMC1.docx]

**SUPPLEMENTAL FILE**

REHAB-HFpEF Trial Organization


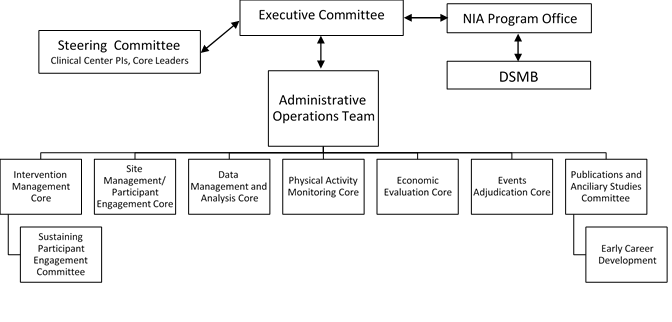


The Coordinating Center is led by the Administrative Operations Team, which oversees the Coordinating Center Cores and Committees. The Executive Committee is the governing group of the trial and is empowered to make high-level decisions which will be implemented by the Coordinating Center. The Steering Committee represents the clinical centers. The NIA and the NIA-appointed DSMB will act in advisory capacities.

**Executive Committee**

The Executive Committee members collectively have relevant expertise (HFpEF, Physical Therapy, Trials Methodology, Biostatistics) to be the governing group of the trial, and will be empowered to make high-level decisions, which the Coordinating Center enacts. The Executive Committee meets weekly, or more often if needed, in order to review progress and efficiently make strategic decisions to address challenges as they arise and any concerns brought by clinical centers, the cores, the DSMB, and NIA.

**Administrative Operations Team**

The Administrative Operations Team is responsible for day-to-day operations and leadership of the trial, administration of the project, and oversees the individual cores that comprise the Coordinating Center. It is responsible for developing and maintaining the protocol, regulatory compliance, reporting, financial tasks, and administering the grant, and is the primary liaison between the NIA Program Office, the DSMB, and the leadership of the trial.

**Site Management and Participant Engagement Core**

The Site Management and Participant Engagement Core is responsible for oversight and engagement of the clinical centers. The Site Management and Participant Engagement Core will be responsible for overseeing and maintaining recruitment, fidelity to the intervention and the protocol, site engagement, and tracking and monitoring of site progress.

**Intervention Management Core**

The Intervention Management Core oversees the delivery and fidelity of the Rehabilitation Intervention. The Intervention Management Core oversees training of the Intervention Leaders at each center using standardized training procedures and develops and maintains the protocol as it relates to the intervention. The Intervention Management Core leads the Sustaining Participant Engagement Committee (SPEC, further details below), and oversees clinical center Intervention Leaders and intervention patients to ensure that the intervention is being delivered in compliance with the protocol. It is also responsible for developing and implementing the comprehensive, tailored adherence and retention strategies designed to facilitate intervention uptake.

**Sustaining Participant Engagement Committee**

The SPEC consists of all clinical center Intervention Leaders and study coordinators. The role of the SPEC is to oversee participant engagement, retention, and adherence at all centers. The SPEC meets biweekly with the Intervention Leaders and site coordinators of all clinical centers to discuss participant engagement issues and participant progression in the intervention. The SPEC also allows for communication between the intervention teams at each site and coordinators to provide feedback about the implementation of the protocol.

**Events Adjudication Committee**

The EAC is responsible for the adjudication of clinical events, including rehospitalizations and deaths, which comprise the primary outcome of REHAB-HFpEF. Adjudication will be managed through a process that includes identification of events, collection of appropriate source documentation, blinded review using event definitions previously utilized in the phase 2 REHAB HF study. Events are reviewed by 2 independent, blinded members of the EAC; the chair of the EAC ensures consensus between the reviewers in contested cases.

**Data Management and Analysis Core**

The Data Management and Analysis Core is responsible for all data management and analysis for the trial, development of the sample size and statistical analysis plans, development of the central tracking system, development of the randomization protocol and procedures, development of the central electronic database. The Data Management and Analysis Core supports the day-to-day operations of many other cores, including being responsible for generating data quality reports for the clinical centers, participant progress reports for the Intervention Management Core and SPEC, enrollment and tracking reports for the Site Management Core, and the DSMB reports. This Core is also responsible for monitoring of data quality control, generating tables and reports, and generating analyses for publications and manuscripts.

**Physical Activity Monitoring Core**

The Physical Activity Monitoring core is responsible for development of the procedures and implementation of the PA monitoring plan. It is responsible for training centers on implementing the accelerometers for real-time monitoring and generates reports to be used by the Intervention Management Core and SPEC.

**Economic Evaluation Core**

The Economic Evaluation Core is responsible for the development and implementation of the costing and data analysis plan. This Core interacts with the Data Management and Analysis Core and Site Management Cores to carry out the collection of economic data, including medical resource use and site-specific provider costs to evaluate the economic impact of the intervention.

**The Publications and Ancillary Study Committee**

The publications committee reviews and approves manuscript and ancillary study proposals derived from this trial. The Publications and Ancillary Study Committee provide recommendations to the Executive Committee, which ultimately provides final approval. In addition, the Publications and Ancillary Study Committee will promote the inclusion of early career investigators in writing groups.

**Data Safety and Monitoring Board**

External trial oversight is conducted by the Program Office of the NIA. The NIA appoints a DSMB to regularly review study progress. The DSMB monitors all aspects of the study, including those that require access to any blinded data. It periodically reviews the progress of the REHAB-HFpEF trial. It is comprised of experts in relevant medical, statistical, operational, and bioethical fields who are not otherwise involved in the study. The DSMB oversees participant safety, evaluate performance, monitor data quality, and provides operational and policy advice to the Executive Committee and coordinating center regarding the status and continuation of the overall study, study components, and clinical centers.

**Advisory Committee**

The Advisory Panel provides the trial with external, high level input, help monitor relevant national and international trends and developments, guide trial progress, and advise on challenges as they may arise. The Advisory Committee is comprised of independent experts in clinical trials and a patient representative.

**Clinical Centers**

The Clinical Centers consist of, at minimum, the site physician investigator, the site Intervention Leader, a study coordinator, and the interventionist(s). The enrolling centers were selected based on the presence of established research infrastructure as well as recognized HF and physical rehabilitation clinical excellence based on their active participation in prior HF trial collaborations. The Clinical Centers are responsible for identification, recruitment of the participants, carrying out the assigned intervention, maintaining follow-up with patients, and maintaining a high degree of protocol fidelity. Each clinical site is responsible for timely reporting of their data into the centralized database for both intervention and trial follow-up. Clinical Centers report to the Site Management Core, and are responsible for carrying out decisions at their site made by the Coordinating Center.

**Clinical Centers and Principal Investigators:**

Advocate Christ Medical Center, Oak Lawn, IL: Nikhil Narang and Anjali Joshi

Atrium Health Wake Forest Baptist, Winston Salem, NC: Dalane W. Kitzman and Olivia Gilbert

Atrium Health Sanger Heart and Vascular Institute, Charlotte, NC: Nicole Cyrille-Superville

Columbia University Medical Center, New York, NY: Mat Maurer

Duke University Medical Center, Durham, NC: Marat Fudim

Henry Ford Health, Detroit, MI: David Lanfear

Inova Heart and Vascular Institute, Falls Church, VA: Chris DeFilippi, Abdulla Damluji

Johns Hopkins Medicine, Baltimore, MA: Kavita Sharma

Medical University of South Carolina, Charleston, SC: Sheldon Litwin

Northwell Health, North Shore University Hospital, Manhasset, NY : Lauren Cooper

Northwestern University, Chicago, IL: Ravi Patel

Novant Health Heart and Vascular Institute, Charlotte, NC: Edward McMillian

Ochsner Health Heart and Vascular Institute, Jefferson, LA: Selim Krim

Thomas Jefferson University Hospital, Philadelphia, PA: Gregory Gibson

University of Alabama at Birmingham, Birmingham, AL: Vera Bittner

University of Michigan Health, Ann Arbor, MI: Scott Hummel

University of Minnesota, M Health Fairview, Minneapolis, MN: Tamas Alexy

University of Pittsburgh Medical Center, Pittsburgh, PA: Daniel Forman

University of Texas Medical Branch at Galveston, Galveston, TX: Wissam Khalife

University of Texas Southwestern Medical Center, Dallas, TX: Ambarish Pandey

VA Pittsburgh Health Care, Pittsburgh, PA: Daniel Forman

Washington University School of Medicine, St. Louis, MO: Michael Rich and Justin Vader

Weill Cornell Medicine, New York, NY: Parag Goyal
