# Supplementary material for: Physical rehabilitation for older patients with acute HFpEF (REHAB-HFpEF) trial: Design and rationale
Source: Am Heart J. Author manuscript; Available in PMC 2026 Apr 15. (PMC13077654; doi:10.1016/j.ahj.2026.107420)
Supplement: MMC2 [file NIHMS2162310-supplement-MMC2.docx]

**SUPPLEMENTAL FIGURE LEGENDS**

**Supplemental Figure 1. Participant Flow diagram by arm with major baseline and follow-up landmarks.**

Participant flow and visit schedule for the REHAB-HFpEF trial. The timing of all study assessments and research contacts is consistent between intervention and attention control arms, mitigating differential exposure to study personnel.

**Supplemental Figure 2. PRECIS-2 Assessment of the REHAB-HFpEF Trial Design** PRECIS-2 wheel summarizing the degree of pragmatism of the REHAB-HFpEF trial across nine domains, demonstrating alignment with real-world clinical practice while maintaining internal validity.

**Supplemental Figure 3. REHAB-HF Intervention Conceptual Model: Mitigating Pathophysiological Pathways and Adverse Outcomes in Acutely Decompensated HFpEF**

The REHAB-HF intervention is designed to counteract the pathophysiological mechanisms and adverse outcomes triggered by hospitalization for acute decompensated heart failure (ADHF) in individuals with heart failure with the preserved ejection fraction (HFpEF) phenotype. Key contributing factors such as multimorbidity, aging, obesity, physical inactivity, and systemic inflammation lead to impairments in capillarity, mitochondrial, and endothelial function and skeletal muscle integrity (sarcopenia). These impairments contribute to frailty and multi-organ dysfunction, including both cardiac and skeletal muscle myopathy, which collectively underlie the clinical syndrome of HFpEF in older adults. Hospitalization for ADHF and the associated immobility (hospital-associated immobility, HAI) further accelerates these maladaptive processes, leaving patients with HFpEF especially susceptible to persistent disability and rehospitalizations. By targeting and mitigating these interconnected pathways, the REHAB-HF intervention aims to improve functional status and quality of life, while reducing rehospitalization, mortality, institutionalization, and healthcare costs.

**
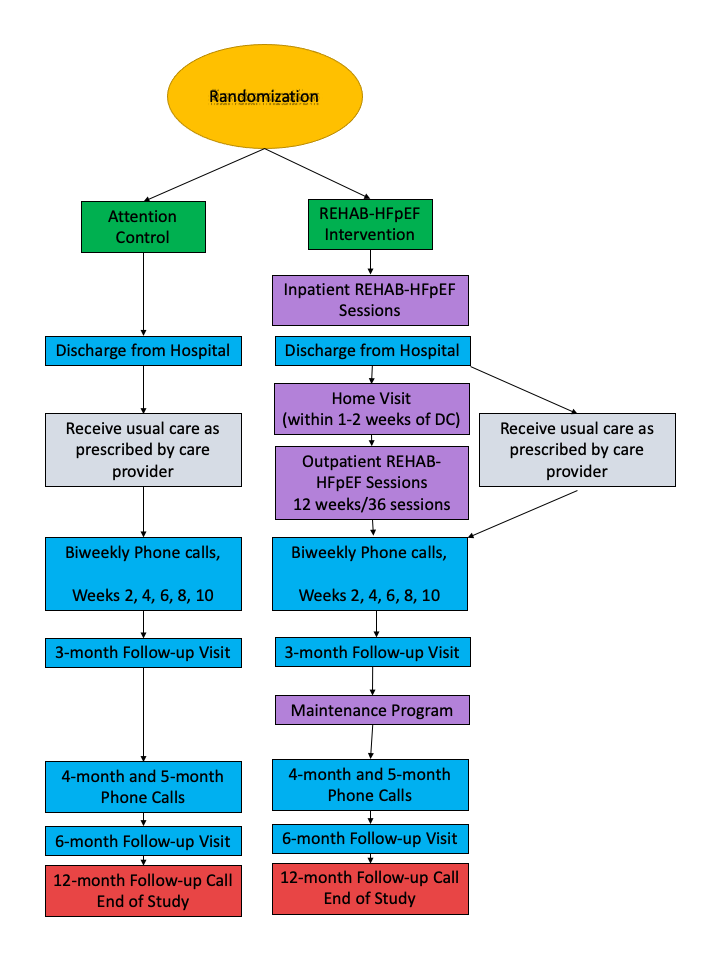
Supplemental Figure 1.**

**Supplemental Figure 2.**

**Supplemental Figure 3.**
